# Effects of transgenic expression of *Brevibacterium linens* methionine gamma lyase (MGL) on accumulation of *Tylenchulus semipenetrans and key aminoacid contents* in *Carrizo citrange*

**DOI:** 10.1007/s11103-017-0666-9

**Published:** 2017-10-20

**Authors:** Elenor Castillo, Federico Martinelli, Florence Zakharov-Negre, Susan E. Ebeler, Tom R. Buzo, Michael V. McKenry, Abhaya M. Dandekar

**Affiliations:** 1 Department of Plant Sciences, University of California, Davis, 1 Shields Ave, Mail Stop 4, Davis, CA 95616-8683 USA; 2 Dipartimento di Scienze Agrarie Alimentari e Forestali, Università degli Studi di Palermo, Palermo, Italy; 3 Department of Viticulture and Enology, University of California, Davis, 1 Shields Ave, Davis, CA 95616 USA; 4 Department of Nematology, University of California, Riverside, 900 University Ave, Riverside, CA 92521 USA

**Keywords:** *Brevibacterium*, Methionine gamma lyase, Nematodes, Sulfur volatiles

## Abstract

**Key message:**

Carrizo transgenic plants overexpressing methionine-gamma-lyase produced dimethyl sulfide. The transgenic plants displayed more resistance to nematode attacks (*Tylenculus semipenetrans*) and may represent an innovative strategy for nematode control.

**Abstract:**

*Tylenchulus semipenetrans* is a nematode pest of many citrus varieties that causes extensive damage to commercial crops worldwide. *Carrizo citrange* vr. (*Citrus sinensis* L. Usb × *Poncirus trifoliate* L. Raf) plants overexpressing *Brevibacterium linens* methionine-gamma-lyase (BlMGL) produced the sulfur volatile compound dimethyl sulfide (DMS). The aim of this work was to determine if transgenic citrus plants expressing BlMGL showed increased tolerance to *T. semipenetrans* infestation and to determine the effect on the content of key amino acids. While transgenic lines emitted dimethyl sulfide from leaves and roots, no sulfur-containing volatiles were detectable in wild-type *Carrizo* in the same tissues. Significant changes detected some key amino acids from leaves of transgenic plants such as aspartate, lysine, glycine, leucine and threonine with no changes in the amounts of methionine and α-ketobutyrate. In roots only glycine showed significant changes across all transgenic lines in comparison to wild-type plants. Transgenic plants expressing BlMGL and emitting DMS had less *T. semipenetrans* aggregation and more biomass than infected WT control plants, indicating that they may represent an innovative management alternative to pesticide/nematicide-based remedies.

**Electronic supplementary material:**

The online version of this article (doi:10.1007/s11103-017-0666-9) contains supplementary material, which is available to authorized users.

## Introduction

Citrus species are common hosts for many nematodes, of which the sedentary semi-endoparasite *Tylenchulus semipenetrans* (citrus nematode) is the most economically important and abundant in agricultural soils. Unlike ectoparasitic nematodes that feed along the exterior of the root, endoparasitic nematodes enter the root tissue and become sedentary where they deliver an arsenal of secretions from esophageal gland cells via the stylet. Practical controls for nematodes are few and consist of amending soil with toxic substances such as anhydrous ammonia (Rodriguez-Kabana 1986) or methyl bromide (Zasada et al. 2010). Although effective, these treatments may be superficial, depending on the depth of the root system. Deep soil penetration of roots beyond the treatment zone causes relatively rapid reinfection. In Florida, uprooting, treating, and moving affected trees to uninfected soil has successfully diminished nematodes numbers; however, this procedure is costly. The emission of volatiles from vegetative root tissues is associated with the plant’s innate defense mechanisms against pests. Certain classes of volatiles such as terpenoids or fatty acid derivatives can deter herbivore feeding (Howe and Jander 2008); however, the herbivore-deterring effects of sulfur volatile compound (SVCs) remain unclear. *Diaphorina citri* Kuwayama, a psyllid that can carry *Candidatus* Liberibacter asiaticus (Las) and cause the disease Huanglongbing or citrus greening, was deterred by volatiles of alliaceous plants more than by volatiles recovered from citrus leaves that did not contain SVCs (Rouseff et al. 2008). Gas chromatography (GC) coupled with detectors specialized to target sulfur volatiles showed that the seed of the Neem tree, *Azadirachta indica* (Meliaceae), produced di-propyl disulfide at levels of 75% of the total volatile profile (Balandrin et al. 1988). Foliar sprays of sulfur have been used for over a century to protect plants against pests and disease (Bloem et al. 2005). Foliar sulfur protects against pecan scab caused by the fungus *Fusicladium effusum* (Wells et al. 2014), leaf spot of oilseed rape infected with *Pyrenopeziza brassicae* (Booth et al. 1991), and powdery mildew caused by the fungus *Erysiphe necator* that affects many fruits grown in humid conditions and moderate temperatures, particularly grape (Kwasniewski et al. 2014). By creating a synthetic version of the methionine-gamma-lyase (MGL) gene from *Brevibacterium linens* and expressing it in *Lactococcus lactis*, a lactic acid bacterium used in milk and cheese processing, food researchers significantly enhanced emission of SVCs for production of sulfur flavor compounds (Hanniffy et al. 2009). Recently, orthologs of the bacterial *MGL* gene were found in protozoa and in *Arabidopsis thaliana* and *Solanum tuberosum*, however, the *Arabidopsis* plants did not emit detectable SVCs, even after a 48-h treatment with an l-methionine solution (Goyer et al. 2007). Two protein-coding genes with 72 and 74% similarity to *At*MGL were present in the potato genome; however, no information is available regarding SVC production in potato (Huang et al. 2012). It is likely that the MGL enzyme is widespread in plants; a nucleotide BLAST search revealed genes with 70–80% identity to *AtMGL* in other plant species (*LOC101263926* in *Solanum lycopersicum, LOC100253026* in *Vitis vinifera*, and LOC102609475 in *Citrus sinensis*).

In this work, we engineered SVC emission in a commonly used citrus rootstock, *Carrizo citrange* vr. (*Citrus sinensis* L. Usb × *Poncirus trifoliate* L. Raf), by overexpressing BlMGL to reduce the typical symptoms caused by infestation of the nematode *T. semipenetrans* in root tissues.

## Materials and methods

### Care of *Carrizo citrange* plants

Wild-type *Carrizo citrange* (*Citrus sinensis* L. Usb × *Poncirus trifoliate* L. Raf.) were grown from seed and *Carrizo* plants newly transformed with MGL were propagated vegetatively in a greenhouse at temperatures between 23 and 35 °C. All plants were potted with UC Davis soil mix and 20-10-20 fertilizer (20% N, 10% P, 20% K) to promote plant growth.

### Construction of plant expression vectors

The 35S:MGL gene was synthesized by ATUM (Newark, California, USA) based on a sequence from plant codon optimization of *Brevibacterium linens* MGL. To develop an *A. tumefaciens*-containing 35S:MGL construct for plant transformation, a chemically synthesized, codon-optimized version of the MGL gene was obtained from ATUM in pJ201:26757. The MGL coding sequence was directionally cloned using XbaI and BamHI into our CaMV35S cassette-containing vector, pDU09.043, such that the coding regions were downstream from the *Cauliflower mosaic virus* (CaMV) 35S promoter and upstream from an octopine synthase gene (*ocs*) 3′-UTR regulatory region required for proper polyadenylation. The resultant cassette carrying MGL gene was inserted into our binary pDU99.2215 vector using AscI. This led to the new binary vector pDU09.043. The binary vector was introduced into a disarmed *Agrobacterium* strain (Dandekar et al. 2012) as described below to create functional plant transformation systems.

### Preparation of *A. tumefaciens* cultures

*Agrobacterium tumefaciens* cells containing pDU09.043 plasmid were grown on LB medium with 0.5 g NaCl, 0.5 g yeast extract, and 1 g tryptone per 100 mL water (pH 6.8 to 7.0). KAN antibiotic was added for specificity to plasmid and bacterial selection. A frozen *A. tumefaciens* stock was used to inoculate a 2 mL starter with LB medium. Initial stock was then transferred to a 250 mL flask containing LB and subcultured for one day. To harvest the *A. tumefaciens*, the culture medium was centrifuged at 4000 rpm for 10 min at room temperature. The *A. tumefaciens* pellet was resuspended in buffer composed of 10 mM MES (500 mM stock at pH 5.6), 10 mM MgCl_2_, and 150 µM acetosyringone in sterile water.

### Agroinfiltration and leaf incubation

The prepared *A. tumefaciens* concentrate (35S:MGL) was diluted in buffer to an optical density of 0.5 at 600 nm and then kept in a darkroom for three hours, after which 0.01% (v/v) Silwet L-77 was added prior to vacuum infiltration. A 50-mL Falcon tube was filled with 40 mL *A. tumefaciens* solution. A harvested *Carrizo* citrus leaf was slightly rolled and subjected to a vacuum treatment up to an absolute pressure of 0.23 atm for 3 min. The infiltrated plant leaves were allowed to air dry for about 15 min. Leaves were transferred to a sealed humidity box and incubated at 20 °C for several days in the dark. Subsequently, tissue was washed in liquid MS medium containing 500 µg/mL carbenicillin for 24 h to kill the bacteria and then plated basal end up in MS medium containing 0.5 mg/L NAA, 0.2 mg/L kinetin and 500 µg/mL carbenicillin. After ~ 10 days, callus from the basal end was removed and transformants were selected using 100 µg/mL KAN. Root growth was promoted using a medium containing 1 mg/L NAA. Subsequently, small seedlings were transplanted into soil pots and acclimatized to growth chamber conditions. DNA was extracted from transgenic plants obtained after acclimation using the Qiagen DNeasy Plant Mini Kit, (Qiagen, Valencia, CA). Plants with the correct-sized PCR amplicon were sent for sequencing to check the presence of MGL transgene. Three MGL-positive plants were sequenced, confirmed and labeled MGL-1, MGL-2 and MGL-6.

### Grafting of wild-type scion in MGL rootstock

BlMGL-expressing lines MGL-1, MGL-2 and MGL-6 served as rootstocks and were grafted with wild-type commercial Valencia orange scions. Wild-type Carrizo rootstock was grafted with Valencia orange scion and used as a control. Grafting was performed with a razor cut in the node region of both plants, which were joined together with rooting powder and sealed with parafilm. Grafting was judged successful after 3–4 months when full development and viable Valencia citrus branching occurred. A 6-mm plug was used to cut 6-mm diameter circles of leaf tissue weighing ~ 2.5 g that were placed in a 20-mL clear glass crimp-top deactivated vial (Restek) for solid phase microextraction (SPME) coupled with gas chromatography sulfur chemiluminescence.

### Amino acid and alpha-keto acid analysis

Twenty mg (± 5) of fresh Carrizo citrus leaf and root tissue was harvested and frozen in liquid nitrogen. Samples were ground by mortar and pestle and placed in an Eppendorf tube. An extraction solution of methanol, chloroform, and water (5:2:2, v/v/v) was degassed with pressurized nitrogen and set in a cooling bath at − 18 to − 22 °C. After grinding, 1 mL pre-chilled extraction solution was added to each sample with care to prevent partial thawing. All samples were chilled on ice, vortexed 10 s, shaken on an Oribital Mixing Chilling/Heating plate for 6 min at 4 °C and centrifuged at 14,000 rcf using a 5415 D centrifuge. The supernatant was removed and divided into two 500-µL portions, one of which was saved as a backup. One 500-µL portion was dried in the Labconco Centrivap cold trap concentrator to complete dryness and then submitted for derivitization (Weckwerth et al. 2004).

### Transcription analysis using RT-qPCR

The first step for RT-qPCR analysis was to extract RNA from leaf and root tissue of transformed *Carrizo* and untransformed, wild-type control plants, performed according to manufacturer’s direction using the Qiagen RNeasy Plant Mini Kit designed for obtaining RNA for cDNA synthesis (Qiagen, Valencia, CA). DNase treatment and cDNA synthesis were completed using protocols described in the manufacture manual QuantiTect Reverse Transcriptase (Qiagen, Valencia, CA). For optimal PCR efficiency, RT-PCR primers for MGL and ELF-1α (reference gene) were designed with the aid of Primer3 (http://www.frodo.wi.mit.edu/cgi-bin/primer3/primer3www.cgi) and are listed in supplemental data.

SsoAdvanced Universal SYBR Green Supermix (Bio-Rad, Richmond, CA) was used with StepOnePlusTM Real-Time PCR 48 well System (Applied Biosystems, So. San Francisco, CA). A five-point standard curve with cDNA amounts ranging from 0.625 to 10.0 ng was built for the MGL and ELF-1α primer sets, with amplification efficiency of 100.6 and 102.5%, respectively. Reactions for leaf samples were analyzed with a total cDNA quantity of 6.25 ng per reaction and three technical replicates and three biological replicates were performed for all. Amplifications were performed using standard amplification conditions: two min at 50 °C, 10 min at 95 °C, and 40 cycles of 15 s at 95 °C and 60 s at 60 °C. Changes in BlMGL gene expression relative to ELF-1α expression were assessed as described (Schmittgen and Livak 2008).

### Solid phase microextraction (SPME) coupled with gas chromatography sulfur chemilluminescence detection

Extraction and analysis of SVCs in wild-type and transgenic *Carrizo* lines were adapted from a previous method for analysis of sulfur-containing volatiles in beer (Miracle et al. 2005). The SPME fiber 50/30 μm divinylbenzene/carboxen/polydimethyl-siloxane (DVB/CAR/PDMS) was exposed for 30 min to the headspace of a 20-mL clear glass crimp-top deactivated vial (Restek) containing either 2.5 g leaf disks obtained from whole leaves with a core borer ~ 6 mm in diameter, or 1.5 g scissor-cut root tissue, at room temperature. Adsorbed volatiles were then desorbed into a 5890 HP GC (Hewlett Packard/Agilent, Little Falls, DE) equipped with a J&W GS-GasPro PLOT column (60 m × 0.32 mm; Agilent, Folsom, CA) connected to a Sievers 355 sulfur chemiluminescence detector (Agilent). The GC was set for splitless injection with an inlet temperature of 250 °C and using a 0.7 mm i.d., deactivated glass SPME injection liner (Supelco, Saint Luis, MO). The split flow was opened 3 min following the injection and closed again 10 min after injection. A volumetric flow of 3 mL/min using helium as a carrier gas was used with a constant column head pressure of 150 kPa. The oven temperature program started with an initial setting of 40 °C followed by an immediate ramp of 10 °C/min to 260 °C, followed by a hold of three min. The SCD burner temperature was 800 °C with a hydrogen flow rate of 100 mL/min and an air flow rate of 40 mL/min. The SCD pressure was six Torr with the controller at 200 Torr.

A SPME fiber blank was analyzed in the beginning of each day to ensure the fiber was clean. Chemical standards (methanethiol, dimethyl sulfide, dimethyl disulfide and dimethyl trisulfide, purchased from Sigma Chemical Co., St. Louis, MO) were extracted and injected periodically as described above for peak identification by retention time. The detector was periodically conditioned as recommended by the manufacturer to avoid loss of sensitivity.

### *Tylenchulus semipenetrans* inoculation

Three MGL-expressing *Carrizo* lines and one wild-type control were tested for tolerance against a population of *Tylenchulus semipenetrans* isolated from the Kearney Ag Research and Extension Center (UCKARE) in Parlier CA. Seven to 13 clones were made for each line used in the analysis. The plants were of uniform size with the same explant initiation date. *Carrizo* plants were placed in 2.5-L plastic pots filled with 2 L soil mixture. The plants were kept in the greenhouse in a completely randomized design at 22 cm^2^ spacing on aluminum tables with an extruded mesh top. Plants were inoculated with 4 g infected soil with ~ 2300 nematodes/g to reach a total of 9300 nematodes per pot.

The ambient temperature mean was 25 °C and the range was 24 to 36 °C after 120 and 240 days. The soil had a mean temperature of 23.6 °C and a range of 13.5 to 37.7 °C after 120 and 240 days. Irrigation was supplied by hand wand every third day with ~ 0.5 L water per pot. More frequent irrigation was applied to individual plants as needed. Miracle Gro was applied at the label rate of 4 g/L and 500 mL solution was used per plant at 75 and 120 days after transplanting. Two foliar sprays of acetamiprid 70% WP with a 0.3 g/L solution were applied to control sucking insects at 30 and 60 days after planting. Weeds were rare and hand-pulled.

Nematode infection was quantified 120 and 240 days after inoculation. Each plant and its roots was separated from the soil by washing with a hand nozzle within a specialized basket lined with a 1 cm^2^ mesh screen to capture breakaway roots. Fresh root biomass was weighed and a 10–20 g diced root sample was placed into a mist-chamber for 5 days with intermittent misting to extract nematodes from within the roots. Extracted nematodes were identified as *T. semipenetrans* and quantified using a dissecting microscope at ×40 magnification.

### Statistical analysis

Statistical data analysis was performed using two-tailed Student t test and Tukey’s test in order to determine significant differences between wild-type and the three transgenic lines for each type of analysis (amino acids, blMGL gene expression, DMS amount, *T. semipenetrans* population, root system biomass and total biomass).

## Results

### Expression of BlMGL in *Carrizo* citrange leaf and root tissue

**Fig. 1:**
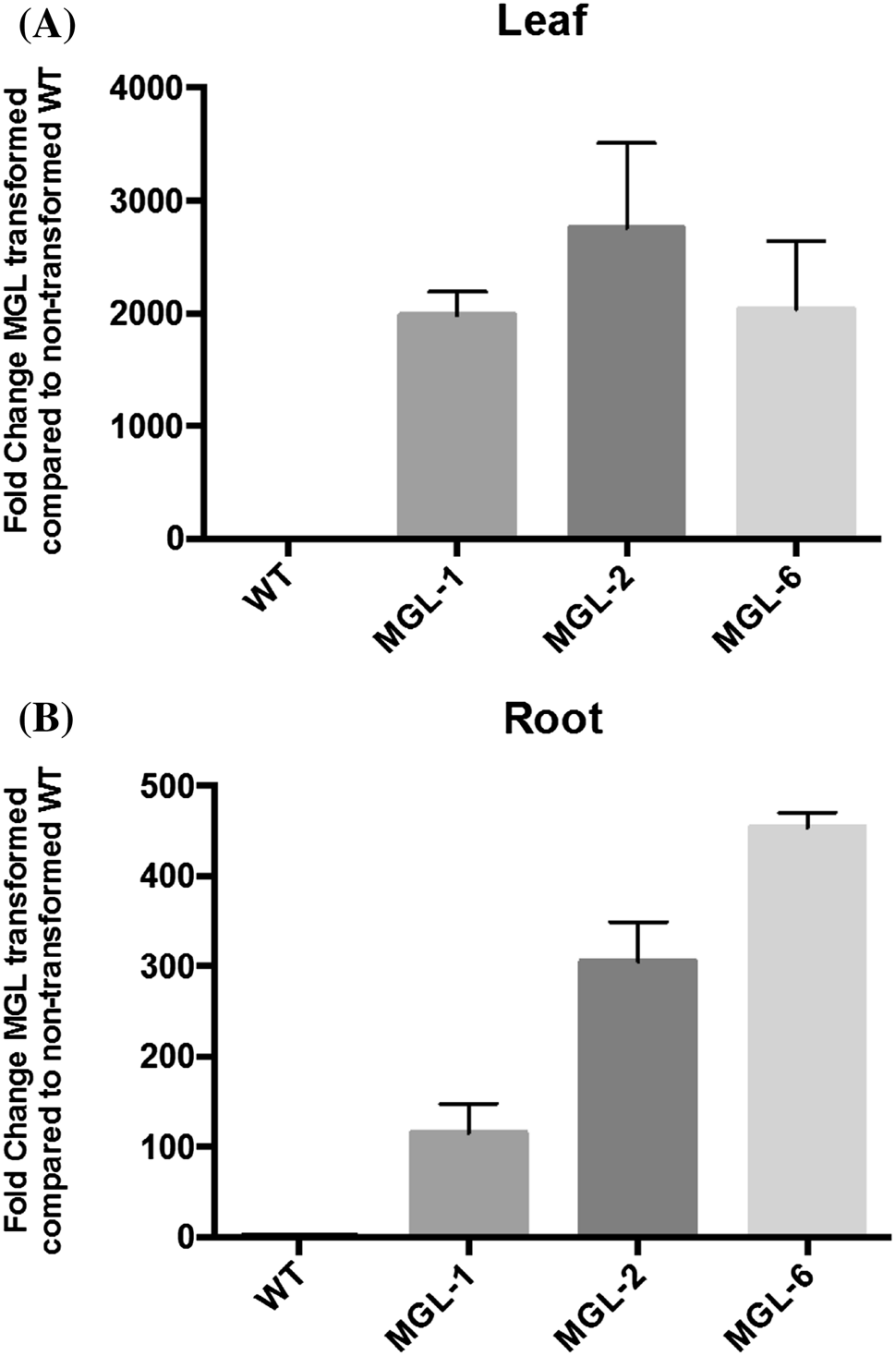
Relative BlMGL gene expression in *Carrizo* **a** leaf and **b** root tissue. Vertical bars represent expression relative to a non-transgenic WT control with a value of 1 (n = 3)

*MGL* expression was detected in leaf and root tissue of transgenic plants, while no expression of *MGL* was observed in non-transgenic WT control plants. *MGL* expression in transgenic citrus leaves was 1981- to 2752-fold greater than in non-transgenic leaves. In root tissue, MGL expression was 115- to 454-fold greater in transgenic lines (Fig. 1).

### BlMGL-expressing lines emit DMS in leaf and root

**Fig. 2:**
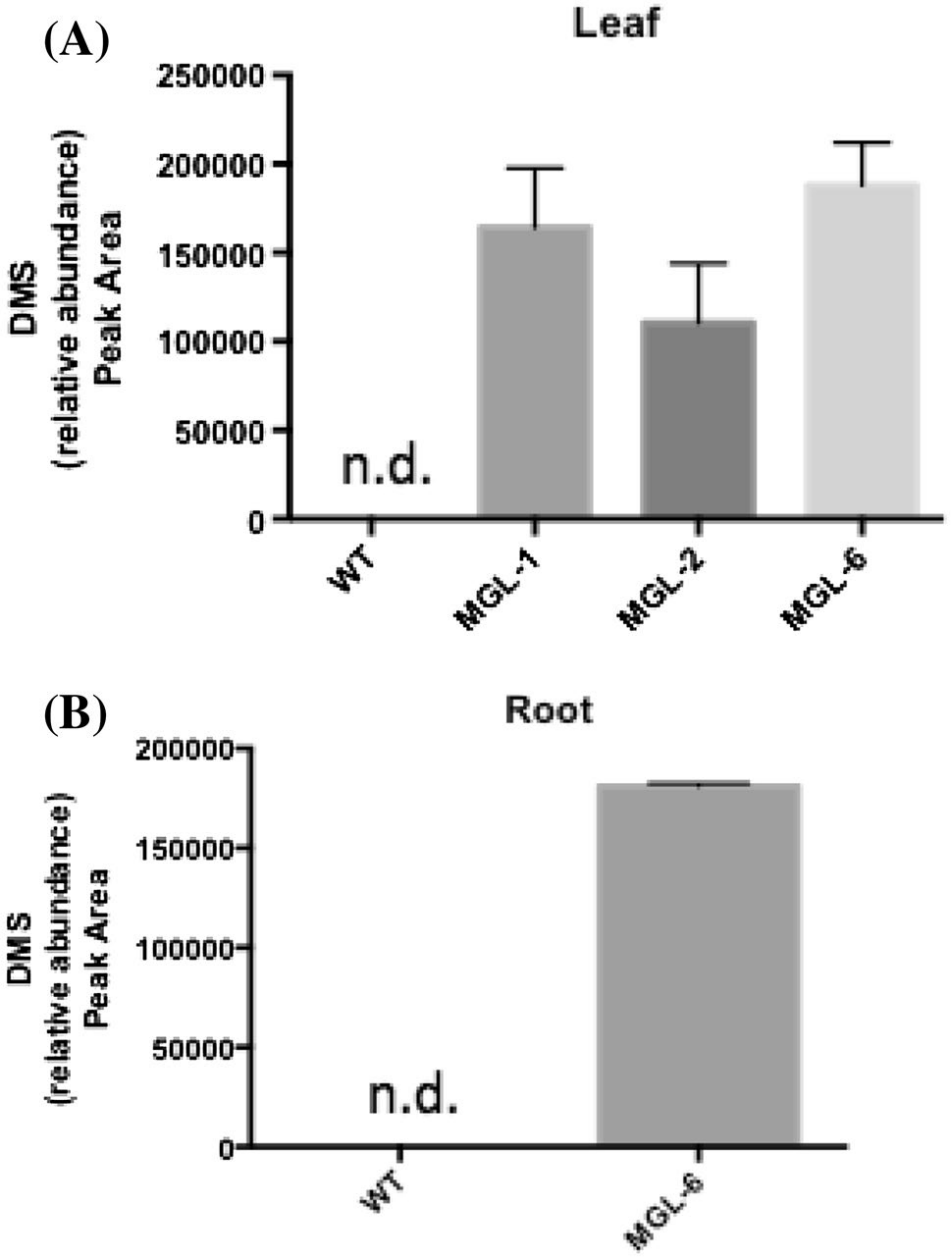
Effect of BlMGL expression on dimethyl sulfide production in *Carrizo* **a** leaf and **b** root tissue. *n.d* not detected; n = 3

All three transgenic lines produced and emitted a single, sulfur-containing volatile, DMS, from leaves, while no sulfur-containing volatiles could be detected in wild type *Carrizo* (Fig. 2a). DMS was the only sulfur-containing volatile detected in roots of line 6 while lines 1 and 2 where erroneously destroyed and unavailable for sampling. No sulfur-containing volatiles were detected in wild type roots (Fig. 2b).

### Analysis of BlMGL expression and the concentrations of selected amino acids and α-ketobutyrate in those tissues

We assessed the impact of introducing BlMGL on the concentrations of selected amino acids and α-ketobutyrate in leaves and roots of transgenic *Carrizo* plants. Since cysteine was under the limit of detection in all lines, cysteine-glycine served as an indicator of cysteine concentration. While some significant differences was observed in the concentrations of some amino acids (aspartate, lysine, glycine, leucine and threonine) in transgenic leaf tissue, there were no consistent patterns allowing us to determine the effect, if any, of BlMGL expression in transgenic lines. Methionine concentrations were not altered in the leaves of transgenic lines, suggesting that BlMGL activity does not deplete the pool of this amino acid in this tissue. Moreover, there were no differences in α-ketobutyrate concentrations, the precursor for isoleucine and a byproduct of the MGL pathway (Table 1).

**Table 1:** Amino and keto acid analysis of leaf tissue from WT and BlMGL-expressing *Carrizo* lines

	WT	MGL-1	MGL-2	MGL-6
Aspartic acid	19,883 ± 343	17,517 ± 2419	18,169 ± 3644	31,332 ± 2412*
Lysine	835 ± 18	630 ± 36*	919 ± 37	2784 ± 405*
Glycine	31,433 ± 5569	6080 ± 810*	5083 ± 1697*	29,058 ± 2647
Leucine	861 ± 61	757 ± 35	1232 ± 94*	12,808 ± 57*
Threonine	3668 ± 264	2740 ± 216	2087 ± 62*	6375 ± 451*
Isoleucine	5292 ± 2960	8251 ± 1180	3110 ± 1880	8328 ± 926
Methionine	131 ± 17	107 ± 23	135 ± 10	139 ± 37
Cysteine–glycine	220 ± 15	399 ± 187	314 ± 71	578 ± 165
α-Ketobutyrate	1.2 ± 0.3	2.0 ± 0.4	1.5 ± 0.1	1.9 ± 0.4

The concentrations of metabolites that differed significantly from the WT are denoted by an asterisk (n = 3, *p* < 0.05, two-tailed Student t test)

While there were significant differences in the concentrations of aspartic acid, lysine, leucine, threonine, methionine and cysteine-glycine in transgenic root tissue compared to WT root tissue, there were no consistent patterns allowing us to determine the effect, if any, of BlMGL expression in transgenic lines and only glycine showed significant differences across all transgenic lines (Table 2).

**Table 2:** Amino and keto acid analysis of root tissue from WT and BlMGL-expressing *Carrizo* lines

Compound average	WT	MGL-2	MGL-4	MGL-6
Aspartic acid	62,678 ± 4544	98,051 ± 13,431	105,960 ± 1901*	61,644 ± 4991
Lysine	11,953 ± 406	8719 ± 1413	10,698 ± 1264	6707 ± 275*
Glycine	5176 ± 524	10,580 ± 1434*	10,978 ± 941*	7450 ± 299*
Leucine	8633 ± 363	2798 ± 352*	8714 ± 573	3563 ± 157*
Threonine	7847 ± 592	3369 ± 463*	6847 ± 126	4407 ± 274*
Isoleucine	7659 ± 1287	7861 ± 1309	6466 ± 3427	3040 ± 1354
Methionine	550 ± 16	356 ± 72	695 ± 42	273 ± 14*
Cysteine–glycine	1112 ± 5	1224 ± 36	786 ± 48*	999 ± 7*
α-Ketobutyrate (ppm)	1.51 ± 0.24	2.12 ± 0.23	2.53 ± 0.43	2.04 ± 0.33

The concentrations of metabolites that differed significantly from the WT are denoted by an asterisk (n = 3, *p* < 0.05, two-tailed Student t test)

### BlMGL expression and the density of *T. semipenetrans* in the *Carrizo* root zone

**Fig. 3:**
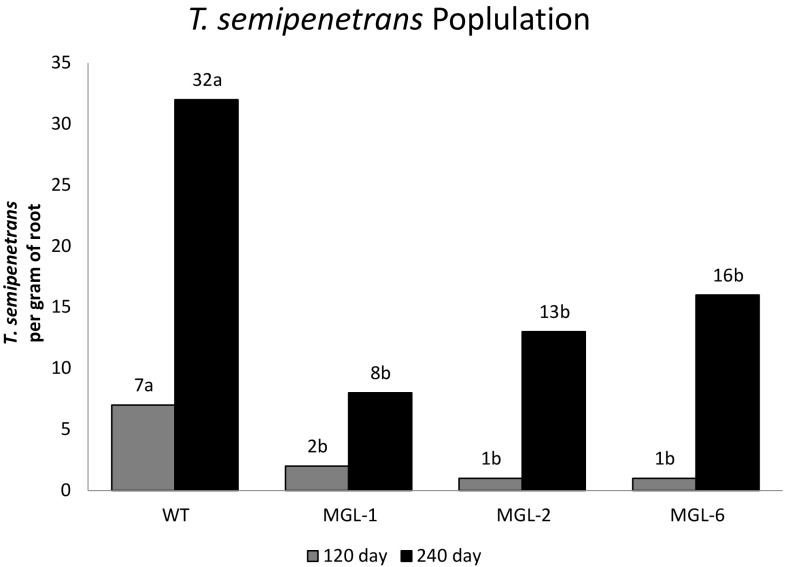
Effect of BlMGL introduction on the infection rate of *Carrizo* roots by *T. semipenetrans*. Bars represent the average (n = 3) nematode count per gram root tissue at 120 days (grey bars) and 240 days (black bars) after inoculation. Statistically significant (*p* < 0.05) differences between WT and BlMGL-expressing lines were evaluated at 120 day and at 240 day by Tukey’s test. Different letters means significant difference between the four plant types at each of the two time points after inoculation

**Fig. 4:**
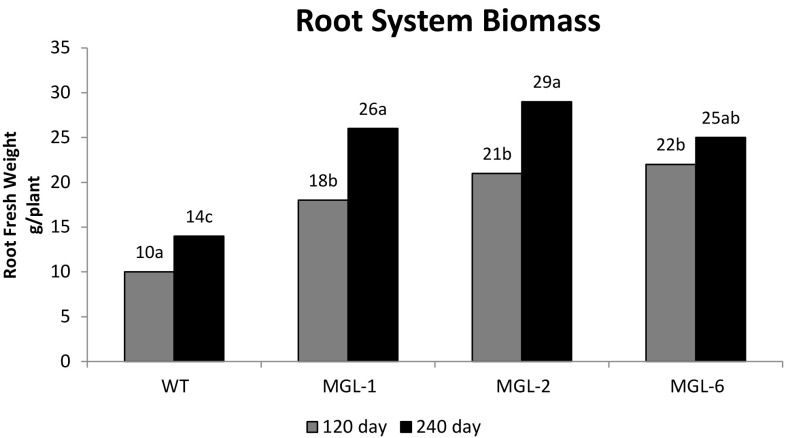
Root system biomass. At 120 days after inoculation (grey bars), statistically significant (p < 0.05) differences ranged from 18 to 22 g per plant for transgenic lines compared to 10 g per plant for WT control. At 240 days (black bars), statistically significant differences ranged from 25 to 29 g per plant compared to 14 g per plant for WT. Bars represent the average (n = 3). A letter indicates statistical differences evaluated by Tukey’s test

**Fig. 5:**
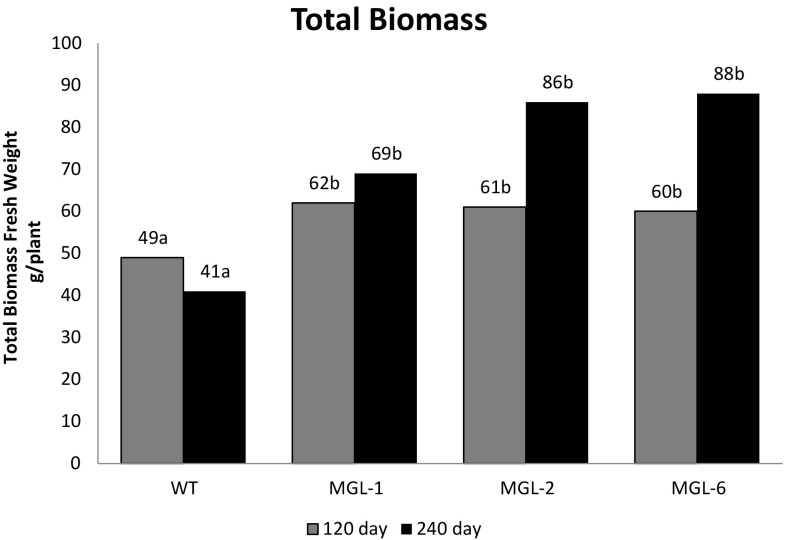
Total biomass fresh weight. Bars represent averages (n = 3) at 120 days (grey bars) and 240 days (black bars) after inoculation. Different letter indicate statistically significant differences (p < 0.05) as evaluated by Tukey’s test; n = 3

No difference was observed between the growth of the transgenic rootstocks grafted with wild-type scion (MGL:WT) and the growth of the wild-type rootstock with wild-type scion (WT:WT) control. No DMS emission were detected in scion leaves 4 months after grafting; suggesting that DMS does not cross the graft junction and emission is localized in transgenic tissues (data not shown). Wild-type and BlMGL-expressing *Carrizo* plants were grown in soil infested with a controlled nematode load. 240 days after inoculation, the *T. semipenetrans* density around the WT roots was significantly greater than in BlMGL-expressing lines (Fig. 3), while root biomass (Fig. 4) and total plant biomass (Fig. 5) were reduced significantly in WT plants compared to BlMGL-expressing lines. Although this trend was also detected 120 days post inoculation, the differences at this sampling time were not statistically significant.

## Discussion

A reduction of nematode presence has been known from a long time to be correlated with the increased concentration of molecular hydrogen sulfide in the soil (Rodriguez-Kabana et al. 1965). The increase of hydrogen sulfide in the soils of rice cultivation suggest that sulfur-related compounds may be effective in control soil-borne pathogens. The fumigation with dimethyl disulfide (DMDS) was effective in controlling some nematode including *T. semipenetrans* in Thomson Seedless grapevines (Cabrera et al. 2014) with no observed phytotoxic effects. DMDS has the benefit to have no negative effects on ozone protection. The action of this compound is on mitochondrial respiration though the block of cytochrome oxidase activity (Auger and Charles 2003).

Our results offer the first evidence that expression of BlMGL *in planta* leads to DMS emission from leaves and roots. We believe DMS emission alone helps to reduce *T. semipenetrans* populations in soil since amino acid analysis offer no consistent patterns amongst transgenic and WT plants and account for reduced nematode populations or reduced plant biomass. These data agreed previous published findings on the use of soil-incorporated different plant material to control soil-borne pathogens (Wang et al. 2009). These authors observed a reduction of *T. semipenetrans* in the soil linked with the production of methyl sulfide and dimethyl sulfide. The incorporation of plant emitting methyl sulfide and dimethyl disulfide decreased the presence of the parasitic nematodes. Our results confimed previous published data that showed how dimethyl disulfide biosynthesized by soil bacteria, could have a reduction effects on nematode population in the soil (Gu et al. 2007). A synergistic action of these sulfide compounds with non-volatile compounds derived by glucosinolate has been hypothesized to increase the control of soilborne pathogens (Wang et al. 2009).

The detection of DMS as the only sulfur-containing volatile compound produced by BlMGL-expressing transgenic *Carrizo* plants was an unexpected result, since previous studies reported DMDS, likely from the auto-catalytic reaction of DMS to DMDS. To our knowledge, DMS emission in plants is rare and only reported in a few halophyte species that produce high concentrations of dimethylsulfoniopropionate (DMSP), the precursor for DMS in these plants (Yoch 2002). However, it is unlikely that *Carrizo* plants started producing DMSP as a result of BlMGL introduction. The sensitive, sulfur-containing volatile detection system used in our study (SPME-GC-SCD) has been used previously for analysis of numerous SVCs in plant tissues and wines (Herszage and Ebeler 2011). It is unlikely that other SVCs were missed by our analysis. In contrast to BlMGL in bacteria, we could not detect MeSH, the precursor to DMS, and we hypothesize that DMS was therefore produced by an unknown, yet efficient, enzymatic thiol methylation of MeSH *in planta*. Thiol methylation activity has been studied in glucosinolate-producing plants, which can form MeSH by methylation of HS- via an S-methyl methionine-dependent halide/bisulfide methyltransferase. The best-studied thiol methyltransferase (TMT) is in cabbage, where thiocyanate, HS- ions and organic thiols can be methylated by TMT. Heterologs of this enzyme were found in 118 species and are thought to be involved in elimination of phytotoxic halide and HS-ions (Attieh et al. 1995). A blast analysis showed a putative thiol methyltransferase in *Citrus sinensis* (accession number XM 006493073.1) with 61% identity at the protein level to the well-characterized cabbage TMT; however, this enzyme has not been characterized and whether these enzymes can use MeSH as a substrate to produce DMS is still unclear. The metabolism of the amino acids isoleucine, threonine, and methionine is interconnected in plants, since both methionine-gamma-lyase and threonine deaminase provide the isoleucine biosynthesis substrate α-ketobutyrate (Joshi et al. 2010). In potato, silencing the two *St*MGLs increased the methionine-to-isoleucine ratio (Huang et al. 2012). This and other studies suggest that MGL plays a subordinate role to production of isoleucine, since α-ketobutryate is a precursor for isoleucine. While there were some differences in threonine concentrations in leaf and root tissues of transgenic lines, there were no clear patterns to determine what effect, if any, BlMGL has on these plants. Surprisingly, we found no change in isoleucine concentrations in leaf or root tissue of transgenic plants compared to WT, which correlated with no significant change in the precursor α-ketobutyrate (Tables 1, 2). Further metabolic profiling is necessary to confirm the impact of BlMGL expression on plant amino acid metabolism. Unexpectedly we did not observe any changes in methionine production due to transgenic expression of BlMGL. This evidence does not agree with the differences observed in enzymatic activities involved in sulphur metabolism occurred in transgenic potatoes (Rinder et al. 2008). Transgenic potatoes with changes in H_2_S have been obtained with possible increased resistance to biotic stresses (Bloem et al. 2011).

The Citrus *Carrizo* genotype is typically used as a rootstock in commercial orchards because it provides resistance to blight, citrus tristeza virus, *Phytophthora* and other nematodes with the exception of *T. semipenetrans* (Niles et al. 1995). Our transgenic, BlMGL-expressing *Carrizo* plants showed tolerance to *T. semipenetrans* and may represent a promising strategy to lessen dependence on pesticidal/nematicidal remedies. Fumigation of cultivated soils for reducing soilborne pathogens is commonly used to obtain a good level of crop quality and yields of many crops in narrow rotation systems (Wang et al. 2009). However, these practices had high detrimental effects such as increasing the production costs, threatening the soil ecosystem and negatively affecting air pollution. Fumigants are not specific compounds targeting a wide range of soil organisms, including those beneficial for crops and they raised health concerns for humans. These suspected effects caused the ban of methyl bromide and an increase of environmental regulations on fumigant compounds. The use of repeated application of metam sodium is required (Di Primo et al. 2003) rendering these practices highly not friendly for the environment. Our results propose a possible transgenic solution for reducing the use of fumigants against nematodes although the public concerns with transgenics will be expected. To decrease the risk of negative impacts of GMOs, such as the uncontrolled flow of transgenes via pollen or seed, we propose a transgenic rootstock strategy that will allow reducing the spread of transgene in the environment. High-throughput proteomic platforms have been used to analyze simultaneously thousands of proteins in plant tissues (Martinelli et al. 2016). This type of approach can be used to analyze scion and rootstock tissues close-by the graft union in order to detect how much transgenic protein may be delivered by the rootstock into the scion.

## Electronic supplementary material

Below is the link to the electronic supplementary material.

Supplementary material 1 (DOCX 11 KB)
