# Supplementary material for: Effects of transgenic expression of Brevibacterium linens methionine gamma lyase (MGL) on accumulation of Tylenchulus semipenetrans and key aminoacid contents in Carrizo citrange
Source: Plant Mol Biol. 2017 Oct 20;95(4):497–505. doi: 10.1007/s11103-017-0666-9 (PMC5688205; doi:10.1007/s11103-017-0666-9)
Supplement: Supplementary file 1 — Supplementary material 1 (DOCX 11 KB) [file 11103_2017_666_MOESM1_ESM.docx]

**Supplementary Data:**

MGL Primers:

MGL RTqPCR F1, oligo 5’-GATTGGGTATTGCTCCCTTT-3’

MGL RTqPCR R3, oligo 5’ ATTACCCTGGTCTTCCTTCG-3’

ELF-1α Primers:

Citrus ELF-1α Fwd 5’ AAGCTGGTATCTCCAAGGATGGT- 3’

Citrus ELF-1α Rvs 5’- CCAAGGGTGAAAGCAAGCA-3’
